# The impact of teleworking in psychologists during COVID-19: Burnout, depression, anxiety, and stress

**DOI:** 10.3389/fpubh.2022.984691

**Published:** 2022-10-03

**Authors:** Carla Serrão, Ana Rita Rodrigues, Andreia Teixeira, Luísa Castro, Ivone Duarte

**Affiliations:** ^1^School of Education, Polytechnic of Porto, Porto, Portugal; ^2^Center for Research and Innovation in Education (inED), Porto, Portugal; ^3^Department of Social and Behavioural Sciences, University of Maia, Maia, Portugal; ^4^Center for Psychology, University of Porto, Porto, Portugal; ^5^Center for Health Technology and Services Research, Faculty of Medicine, University of Porto, Porto, Portugal; ^6^Department of Community Medicine, Information and Health Decision Sciences, Faculty of Medicine, University of Porto, Porto, Portugal; ^7^ADiT-LAB, Instituto Politécnico de Viana do Castelo, Viana do Castelo, Portugal; ^8^School of Health, Polytechnic of Porto, Porto, Portugal

**Keywords:** COVID-19, telework, burnout, depression, cross-sectional analysis

## Abstract

**Background:**

The COVID-19 pandemic has forced mental health professionals to adapt quickly. The pandemic has created multiple new tasks for the psychologist. In addition to the various stressors closely linked to the COVID-19 pandemic, psychologists were forced to make their services more flexible. Teleworking was a way of continuing to work.

**Objective:**

This study aimed to identify the impact of working pattern on the levels of burnout, depression, anxiety, and stress.

**Methods:**

This was a cross-sectional study based on an online questionnaire applied to eighty-three Portuguese psychologists. Data were collected from May 9 to June 8, 2020, a period comprising the declaration of a national calamity and then state of emergency, and the subsequent ease of lockdown measures. The Copenhagen Burnout Inventory Scale and Depression Anxiety and Stress Scale were used. Univariate multiple linear regression models were estimated for each mental health outcome.

**Results:**

Significant differences were found between psychologists working in the workplace and in teleworking at the personal burnout, work-related burnout, client-related burnout, depression, and stress. In multiple linear regression, teleworking, not working, and being unmarried was significantly associated with higher levels of depression. Teleworking was significantly associated with higher stress scores and client-related and work burnout.

**Conclusions:**

This exceptional time of sudden, mandatory, and high-intensity teleworking, required rapid adaptation, giving rise to new stressors that might have been responsible for burnout levels in psychologists.

## Introduction

The World Health Organization (1) declared the current outbreak of COVID-19 a pandemic on March 11, 2020. On 13 March 2020, the portuguese government declared a state of alert, which forced the closure of schools and businesses to contain the spread of the SARS-CoV-2 vírus.

This global outbreak created a significant set of challenges, notedly for the psychologists and other mental health professionals, as it affected mental health and aggravated previous psychopathologies (2). In fact, in the pandemic event, “mental health workforce has been forced to rapidly adapt by using teleconferencing, telephone, and other telehealth modalities to deliver psychological care” (3) (p. 175). The Eurofound (4) survey found that close to 40% of those currently working in the EU began to telework fulltime because of the pandemic. In Portugal telework became obligatory on March 19 until early July. Despite the mandatory nature of telework, all the rules of the Portuguese Labor Code remained the same, that is, the maximum load for all sectors is 40 h per week, distributed in 8-h days, including breaks.

Teleworking or telecommuting “involves working at a remote location away from a central office” (5) (p. 44). But telework conditions during COVID-19 are not the same as for teleworking under normal circumstances. Teleworking was a forced-choice and was developed as full-time. Also, it is likely that other family members will be at home at the same time (6), and this can create difficulties for work-family balance (1), increased guilty feelings about neglecting issues at home (7). Besides that, and according to Hamouche (8) (p. 12) “the level of stress may increase with the presence of children at home since schools are closed.” The data provided by the Eurofound survey (2020) indicate that among those working from home: “26 per cent live in households with children under 12, and another 10 per cent are living with children aged 12–17. These workers find balancing their work and care responsibilities challenging and are experiencing new dynamics in managing their work-life balance.”

The amount of research on the topic of telework during the COVID-19 pandemic is limited. However, pre-pandemic studies [e.g., (9)] showed that telework enhances the quality of life by improving the processes for reconciling family life and working life. Also, teleworking reduces the stress and strain associated with home-work-home travel (9). Greater time flexibility (10) and more autonomy and comfort have been reported as advantages of teleworking, according to the worker's perspective (11). Despite these potential benefits, some evidence indicates that telework leads to increase stress (12) and anxiety. Teleworkers can work longer hours because the boundaries between personal and professional life are more blurred (13). Telework can create difficulties to separate personal and family matters from professional issues, a potential increase in household responsibilities when working from home, and trigger social and professional isolation (5).

The ongoing pandemic has created multiple new tasks for the psychologist. In addition to the various stressors (e.g., the perception of security and the threat and risk of contagion; quarantine and confinement; possible financial loss and job insecurity) closely linked to the COVID-19 pandemic [e.g., (14, 15)], psychologists were forced to make their services more flexible. Many of these professionals had to learn and develop new skills (3) and face additional challenges, like insecure network connections, distractions in the home environment, and securing platforms that do not present any additional security or confidentiality measures (16). At the same time, these professionals face their own uncertainties, anxiety, fears, and difficulties (16).

Dealing with this set of demands can cause burnout in psychologists. Burnout is “a state of physical, emotional and mental exhaustion that results from long-term involvement in work situations that are emotionally demanding” (17) (p. 501). Burnout is “the degree of physical and psychological fatigue and exhaustion experienced by the person” (18) (p. 197). Part of this increase in burnout could be explained by the unique nature and requirements of the mental health profession, such as intensified emotional labor with patients, a higher volume of paperwork (19), being on call frequently or the perception that patients are not improving (16). If we add the COVID-19 context and teleworking to this set of stressors, we might be facing a problem of mental health. Although there is an extensive body of literature on burnout syndrome, a small number of investigations are related to psychologists.

Despite this hypothesis, burnout research has focused on traditional workers, but those studies have been slow to extend their interest to teleworkers.

In addition, under normal circumstances, teleworking in Portugal is not very common, with only 6.5% of workers working from home (20). Faced with this unprecedented issue, the objective of this work was to identify the impact of working pattern (telework, work in the workplace, and not to work) following COVID-19 restrictions, at the level of burnout, depression, anxiety, and stress in a sample of Portuguese psychologists.

## Materials and methods

### Participants

This study consisted of psychologists, who were Portuguese speakers, and were working or unemployed in Portugal at the time COVID-19 pandemic started. No other eligibility criteria existed.

Eighty-three psychologists (Mage = 38.2; SD = 9.5) participated in the present study. The power associated with the sample size obtained was computed using G^*^Power version 3.1.9.7 (21). For an ANOVA one-way test, a significance criterion of α = 0.05, an effect size of 0.35 (medium-large) and a sample size of 83 participants, the power achieved was 0.81.

Nearly all were women (84.3%), and 49.4% were married. Of the forty (48.2%) participants who had children, 72.5% had children aged 12 years old or less. A total of 16 (19.3%) participants were caregivers of older people or people with disabilities. Psychologists who were not working accounted for 14.5% of the sample, 10.8% (*n* = 9) had their activity suspended, 2.4%; (*n* = 2) were in maternity leave or childcare; and 1.2% (*n* = 1) were unemployed. The demographics and professional characteristics of the 83 participating psychologists are summarized in Table 1.

**Table 1:** Demographic and professional characteristics of psychologists (*n* = 83).

**Characteristics**	***N***	**%**
**Sex**		
Female	70	84.3
Male	13	15.7
**Working pattern**		
Teleworking	50	60.2
Working in the workplace	21	25.3
Not working	12	14.4
**Death of relative or friend during the pandemic**		
Yes	6	7.2
No	77	92.8
**Education level**		
Graduate	23	27.7
Master	54	65.1
Doctorate degree	6	7.2
**Years of professional experience**		
Five years or less	29	34.9
From 6 to 10 years	11	13.3
From 11 to 15 years	15	18.1
More than 15 years	28	33.7
**Salary reduction**		
Yes	34	41.0
No	49	59.0
**Diagnosed health problem**		
Yes	19	22.9
No	64	77.1
**COVID-19 tested**		
Yes	10	12.0
No, I have no interest	24	28.9
No, but I would like to do it	49	59.0

Almost the entire sample indicated not working directly on diagnosis and/or treatment of persons infected with COVID-19 (94%; *n* = 78).

### Measures and instruments

The survey included sociodemographic data (e.g., sex, age, civil status, information regarding the existence of children, academic status, years of clinical practice, area of residence, work setting before COVID-19, work status during COVID-19). To analyse psychological variables, the Copenhagen Burnout Inventory and the Depression Anxiety and Stress Scale were used.

Copenhagen Burnout Inventory (CBI) (18)—This is a 19-item tool integrating three subscales: personal burnout (six items), work-related burnout (seven items), and client-related burnout (six items). Personal burnout is described as “...the degree of physical and psychological fatigue and exhaustion experienced by the person” (18) (p. 197). Personal burnout might also occur among those who do not work (e.g., unemployed, early retired people, pensioners, and housewives). That is, according to the authors of the CBI, people who are not working can also experience the exhaustion and fatigue that is typical of workers. The work-related burnout assesses the symptoms that respondents attribute to work. The client-related burnout describes feelings of physical and psychological fatigue and exhaustion that respondents attribute to their work with patients. The score for each subscale is the average of item scores within the subscale, and ranges from 0 to 100. Scores of 50 or above in each of the three subscales were considered high-level burnout. These subscales are characterized by high internal consistency (original version: α = 0.84 and Portuguese version: α = 0.86, where α is the Cronbach's alpha). In the current study, the Cronbach's alphas obtained were 0.91, 0.89, and 0.86 for personal burnout, work-related burnout, and client-related burnout, respectively.

The Depression Anxiety and Stress Scale (DASS-21) (22) was used to evaluate mental health symptoms. This version consists of 21 items, which include three self-report subscales designed to measure the negative emotional states of depression, anxiety, and stress. Each of the three subscales contains seven items, and the respondents are asked to rate the extent to which they have experienced each state over the past week, using a 4-point Likert scale: 0 = Did not apply to me at all; 1 = Applied to me to some degree, or some of the time; 2 = Applied to me to a considerable degree or a good part of the time; and 3 = Applied to me very much or most of the time. The scores for each subscale vary from 0 to 21, with higher scores indicating a more negative emotional state. In the current study, Cronbach's alphas obtained were 0.80, 0.83, and 0.89 for depression, anxiety, and stress, respectively.

### Procedure

This was a cross-sectional quantitative web-based study applied to Portuguese psychologists, spread *via* social networks using a snowball technique, and supported by health care institutions and professional organizations. Data collection was performed on a Google Forms platform available between May 9 and June 8, 2020, a period included in the national calamity declaration and easing of lockdown measures that followed a state of national emergency (between March 18 and May 2). It was conducted in line with the Declaration of Helsinki and received approval from the Faculty of Medicine of University of Porto's Ethics Committee (Ref 184/2020 on May 7, 2020). All participants gave their online informed consent at the beginning of the survey; when accessing the link participants were presented with an introduction with the study purposes, duration of the survey, and guarantees of anonymity and confidentiality. If they agreed with the study procedures, they were asked to click on a confirmation button to proceed to the survey.

### Data analysis

Data analysis was performed using SPSS v.26 (IBM SPSS Inc.)^®^. Categorical variables were described by absolute and relative frequencies, *n* (%). Normally distributed quantitative variables were described by the mean and the respective standard deviation, M ± SD. Quantitative variables not normally distributed were described by the median and the interquartile interval, Med (Q1; Q3). The normality of quantitative variables was verified by observation of histograms. The comparison of quantitative variables between two groups was made by the Student *t*-test, if the variables were normally distributed or, otherwise, through the Mann-Whitney test. The comparison of quantitative variables between more than two groups was made by the One-way ANOVA test, if the variables were normally distributed or, otherwise, through the Kruskal-Wallis test; in both cases, if there were differences, multiple comparisons were performed using Bonferroni adjustments. For the psychological variables (outcomes) where there were significant differences between working patterns (working on the workplace; teleworking; not working), a separated multiple linear regression was performed. To decide which independent variables to include in each multiple regression, simple linear regressions were performed with each of the following variables: years of professional experience (≤5 years; 6–15 years; >15 years), marital status (married; single/divorced/separate/widowed), and children (≤12 years old; no children or >12 years old). All variables that correlated with the outcomes at *p* ≤ 0.05 in a simple regression were included in the multiple linear regressions. Only the significant variables were maintained in the final multiple models. The results of linear regressions were presented by the estimated coefficients (β), the respective 95% confidence interval (95% CI), and *p*-value. To evaluate the model, the F statistic of the overall model test, the respective *p*-value, and the value of the determination coefficient (*R*^2^) were presented. Assumptions of linear regressions were verified by visual observation of histograms, for normality of residuals and variance homogeneity of residuals were verified by visual inspection of scatter plots. Values of *p* ≤ 0.05 were considered significant.

## Results

### Sample characteristics of participants

The average levels of burnout in psychologists were grouped according to high and low burnout. Among participants, 31 (37.3%) had high work-related burnout, 28 (33.7%) had high personal burnout, and 14 (16.9%) high client-related burnout.

For depression, anxiety and stress, participants obtained a median (Q1; Q3) score of 1 (0; 3), 1 (0; 3), and 5 (3, 8), respectively. It can thus be concluded that this sample presented levels of depression, anxiety, and depression well-below the midpoint of the subscales.

It should be noted that, based on these results, the symptom of stress seems to be the most experienced by this sample, although it departs significantly from values considered worrying from the point of view of mental health.

Regarding personal burnout and once this dimension has the objective of assessing the fatigue or exhaustion of individuals, regardless of their occupational status (including unemployed, pensioners, etc.), a comparison was made between the three groups (working in the workplace; teleworking; not working). Using the Kruskal Wallis test, results indicated significant differences between the three-working patterns (*p* = 0.022; see Table 2). Psychologists in telework showed a higher level of personal burnout (Med = 45.8) compared to the group that was not working (Med = 35.4) and the group that was working in the workplace (Med = 25).

**Table 2:** Burnout, depression, anxiety, and stress levels comparison among three groups of psychologists: those who have been working in the workplace (*n* = 21), those who have been teleworking (*n* = 50), and those who have not been working (*n* = 12).

**Variables**	**Working in the workplace** **med (Q_1_; Q_3_)**	**Teleworking** **med (Q_1_; Q_3_)**	**Not working** **med (Q_1_; Q_3_)**	***P*** **-value**
Personal burnout	25 (18.8; 43.8)	45.8 (32.3; 55.2)	35.4 (18.8; 59.4)	0.022^a^, ^*^
Work-related burnout	39.3 (25; 48.2)	42.9 (35.7; 53.6)	–	0.024^b^, ^*^
Client-related burnout	8.3 (2.1; 20.8)	29.2 (16.7; 41.7)	–	<0.001^b^, ^*^
DASS-depression	0 (0; 2)	2 (0; 4.3)	1.5 (0.3; 4.8)	0.025^a^, ^*^
DASS-anxiety	0 (0; 3)	1 (0; 3.3)	1 (0; 5)	0.543^a^
DASS-stress	3 (1; 5.5)	6 (4; 10.3)	5.5 (1, 11)	0.015^a^, ^*^

a Kruskal-Wallis test.

b Mann-Witney test.

* significant at 5%.

Work-related burnout and client-related burnout were compared only between the groups that were working. Psychologists in telework reported significantly higher levels of work-related burnout (Med = 42.9) and client-related burnout (Med = 29.2) than psychologists who were working in the workplace (Med = 39.3 and Med = 8.3, respectively).

Regarding depression, anxiety, and stress subscales, differences were found between the groups only in depression and stress (*p* = 0.025 and *p* = 0.015, respectively). Again, psychologists in telework showed higher levels of depression (Med = 2) and stress (Med = 6) when compared to psychologists who were not working during the COVID-19 outbreak (Med = 1.5 and Med = 5.5, respectively) and to those who were working in the workplace (Med = 0 and Med = 3, respectively; see Table 2).

Multiple comparisons were made for the scales in which there were differences between the three groups. *Post-hoc* tests were conducted to compare working in the workplace or teleworking or not working on the dependent variable's personal burnout, depression, and stress (see Table 3). The results show a significant difference between the group that was working in the workplace and the group that was teleworking. Psychologists in telework present significantly higher levels of personal burnout (45.8 vs. 25; *p* = 0019), depression (2 vs. 0; *p* = 0.026), and stress (6 vs. 3; *p* = 0.011), compared to psychologists who developed their activity in the workplace.

**Table 3:** The *p*-values of multiple comparisons (Bonferroni adjustments).

**Variables**	**Working in the workplace vs. Teleworking**	**Teleworking vs. not working**	**Not working vs. working in the workplace**
Personal Burnout	0.019^*^	0.969	0.831
DASS-depression	0.026^*^	1.000	0.163
DASS-stress	0.011^*^	1.000	0.507

* significant at 5%.

### Results of personal burnout, work-related burnout, client-related burnout, and dass-subscales: Univariate multiple linear regressions

For each outcome—personal burnout, work-related burnout, client-related burnout, depression, and stress—a separated simple linear regression was performed considering work pattern as an independent variable. To adjust for possible confounders, separated simple linear regressions were performed considering the following socio-demographic variables as independent variables: years of professional experience (≤5 years; 6–15 years; >15 years), marital status (married; single/divorced/separate/widowed), and children (≤12 years old; no children or >12 years old). Notice that for work-related burnout and client-related burnout, the psychologists who have not been working were excluded (*n* = 12). All variables that correlated with the outcomes at *p* ≤ 0.05 in a simple regression were included in the multiple linear regression (see Table 4).

**Table 4:** Regression coefficients, from univariate multiple linear regressions.

**Variables**	**Personal burnout (*n* = 83)** **β [95% CI]**	**Work-related burnout (n = 71)** **β [95% CI]**	**Client-related burnout (n = 71)** **β [95% CI]**	**DASS-depression (*n* = 83)** **β [95% CI]**	**DASS-stress (*n* = 83)** **β [95% CI]**
**Working pattern**					
Working in the workplace	Reference		Reference	Reference	Reference
Teleworking	11.5 [1.6; 21.4]^*^	NI	16.3 [7.8; 24.9]^*^	1.6 [0.3; 2.9]^*^	3.2 [1.0; 5.3]^*^
Not working	4.8 [−9.0; 18.6]		NI	1.9 [0.10; 3.7]^*^	2.1 [-1.0; 5.1]
**Parental status**					
No children or >12 years old	Reference	Reference	NI	NI	NI
<12 years old	10.8 [2.0; 19.6]^*^	11.6 [3.6; 19.6]^*^			
**Marital status**					
Married single/divorced/separate/widowed	NI	NI	NI	Reference 1.5 [0.4; 2.7]^*^	NI
*R* ^2^	0.141	0.107	0.174	0.155	0.094
F; *p*-value	4.33; 0.007	8.29; 0.005	14.5; <0.001	4.83; 0.004	4.16; 0.019

NI, not included.

* significant at 5%.

Researchers often have reported on the effects of demographic factors on burnout. For example, Cordes and Dougherty (23) indicated that married individuals experienced less burnout than those who are single. The metanalysis study led by Brewer and Shepard (38) concluded that there was a small negative correlation between years of experience and exhaustion.

### Personal burnout

Teleworking and having children under 12 years old were significantly associated with higher levels of personal burnout. Participants in teleworking had an 11-point rise (β = 11.5, *p* = 0.024) in the average level of personal burnout in comparison with participants who worked in the workplace. Having children under 12 years was associated with higher personal burnout levels in comparison with not having or having children over 12 years old (β = 11.5, *p* = 0.017). In this model, the independent variables explained 14.1% of the variability of the personal burnout in the sample.

### Work-related burnout

Having children under 12 years was associated with increased levels of work-related burnout (β = 11.6, *p* = 0.005). This variable explained 10.7% of the variability in the work-related burnout.

### Client-related burnout

Teleworking was significantly associated with higher client-related burnout (β = 16.3, *p* < 0.001). This variable explained 17.4% of the variability in the client-related burnout in the sample.

### Depression

Teleworking was associated with an increase in depression average levels (β = 1.6, *p* = 0.016). Also, for psychologists, not working was associated with higher levels of depression (β = 1.9, *p* = 0.039). Single status showed significantly more depression when compared to marriage status/non-marital partnerships (β = 1.5, *p* = 0.007). In this model, the independent variables explained 15.5% of the variability of depression in the participants.

### Stress

Teleworking was identified as significantly associated with higher stress levels (β = 3.2, *p* = 0.005). This variable explained 9.4% of the variability in stress.

## Discussion

Through a cross-sectional survey, this study aimed to identify the impact of working pattern (telework, work in the workplace, and not to work) following COVID-19 restrictions on the levels of burnout, depression, anxiety, and stress in a sample of psychologists.

According to Kristensen et al. (18) (p. 197), burnout is “the degree of physical and psychological fatigue and exhaustion experienced by the person.” The exhaustion develops across different life domains (e.g., personal sphere, work experience, and interaction with clients). In this study, about a third of psychologist's (37.3%) assign physical and psychological exhaustion as related to work (18). This result can be explained by the nature of the profession, as well as the rapid and dramatic change in the provision of services resulting from teleworking (5, 24).

Regarding the remaining dimensions of burnout, the results indicate that 33.7% had high personal burnout, and 16.9% had high client-related burnout. These finds are in line with other studies [e.g., (25, 26)], where it was found that the highest levels of burnout were those related to work and the lowest were those related to the client [e.g., (25, 26)]. Also, the burnout prevalence observed in this study was higher than in previous studies in Portuguese psychologists (pre COVID-19), showing that 25% reported emotional exhaustion and 7.7% cynism (unfeeling and impersonal response toward patients) (27) and 27.3% reported global burnout (28). Nerveless, these studies assessed burnout with a different instrument (Maslach Burnout Inventory) and direct comparisons are difficult. In addition, these studies were restricted to Autonomous Region of Madeira (27) and to Autonomous Region of Madeira and Azores (28).

Although the percentage of psychologists who showed burnout associated with the client was low (16.9%), teleworkers exhibited much higher levels of burnout compared to psychologists who were working in the workplace. The pattern of client-related burnout could be symptomatic of compassion fatigue (defined as secondary traumatic stress disorder) and compromising the psychotherapist's ability to experience the painful emotions of the client (29). Several unprecedented factors might contribute to this finding: psychologists having to deal with the same problems, fears and concerns as their clients; challenges of social isolation; not having supervision or the possibility to discuss cases with colleagues (16), might justify this result.

These results are worrying, whereas higher levels of burnout compromise the capacity of professionals to take care of themselves and their clients. Besides, burnout can also mitigate the ability to provide empathy, support, and guidance in their therapeutic work, thus compromising the client's progress and wellbeing (30).

Being at telework and having children under 12 years old were associated with higher levels of personal burnout. If we consider these variables and the fact that most of our sample are women, we can hypothesize that achieving a balance between family and work is challenging. Recently, in the context of pandemic restrictions, the results of the survey conducted by Lean In and Survey Monkey (31) indicate that women have been much more affected by work-family stress than men.

Psychologists who had an infant or children under 12-year-old were more likely to experience personal burnout and work-related burnout. This is not surprising as, during the COVID-19 crisis, telework often must be combined with taking care of the children (due to the closure of schools and day-care facilities).

Portuguese psychologists in teleworking and with suspended activity showed higher levels of depression. Under the assumption that teleworking, by definition, reduces physical and personal interaction (32), negative effects might have emerged in terms of the experience of social and professional isolation (5). Depression can be the result of intense stress that has not been managed (8), and financial loss and job insecurity might be considered as long-lasting stressors related to COVID-19 (8).

Being not married was associated with higher levels of depression in comparison with married psychologists. It was not surprising that we did not find differences in burnout between married and unmarried people, since being married provides a support network that can be helpful in buffering the debilitating impact of occupational stress/ burnout (33).

The identification of who might be more affected by COVID-19, not epidemiologically but simply by working patterns, has important implications for mental health professional practice and supervision. Our research insights can contribute to better organizational management of the challenges that psychologists face in teleworking, and consequently, they can help create preconditions for positive adaptation to the telecommuting work environment.

It is essential, in the first instance, to recognize that teleworking involves not only merging the work and personal spaces, being far from the usual social and work dynamics, but also having to simultaneously manage unusual family dynamics (1).

In the second instance, we must be aware that working remotely does not mean working alone. On the contrary, valuing teamwork becomes even more important in circumstances like this. That said, it is essential to share experiences, difficulties, and challenges between peers, establishing a connection and creating a feeling of mutual support. By strengthening this contact between peers, we will also be moving toward reducing social and professional isolation (1), which, as we have seen, are factors that contribute to the exacerbation of stress, anxiety, depression, and burnout of this professional class.

Another crucial aspect that we want to highlight here is the importance of self-care. The term self-care refers to the “engagement in behaviors that maintain and promote physical and emotional wellbeing (34) (p. 56). When taking care of themselves, psychologists will also be better able to take care of others. Engagement in self-care is related with greater wellbeing (35), higher levels of positive affect, flourishing (36), and compassion satisfaction (37).Therefore, it is important to respect the moments of breaks (e.g., days off, weekends) to relax or perform a rewarding activity, as well as maintaining a physical exercise routine and respecting eating and sleeping routines (1). Also, the practice of mindfulness can be useful, because, in addition to helping in the management of stress and anxiety, it allows these professionals to be aware of their experience, as well as their physical and mental limits and needs, without identifying themselves too much in the most challenging/distressing moments.

### Limitations

This study has several limitations. First, it has a cross-sectional design, which does not allow for causal conclusions. Second, this was a cross-sectional online survey, which might have limited the accessibility of people less familiar or less prone to use the internet. Third, psychologists with high levels of burnout might have been less likely to participate in this study. Fourth, this study was unable to distinguish pre-existing mental health symptoms from new symptoms. Another limitation refers to the fact that the characteristics in which the telework was developed were not investigated, i.e., number of working hours per day in teleworking compared to usual; equipment used at home and working conditions, etc. We assumed, according to the Portuguese Labor Code, that the same rules were kept, i.e., up to 40 h per week. In this sense, in future studies variables associated with telework characteristics should be investigated.

## Conclusion

In summary, the present study, conducted during the first wave of the COVID-19 pandemic (between May 9 to June 8, 2020, a period comprising the declaration of a national calamity), found that 37% had high work-related burnout, 34% had high personal burnout, and 17% high client-related burnout. This exceptional time of sudden, mandatory, and high-intensity teleworking, required rapid adaptation, giving rise to new stressors that might have been responsible for burnout levels in psychologists. Emotion management strategies and self-care should be recommended. Mindfulness based interventions can be useful in the management of stress and anxiety.

## Data availability statement

The raw data supporting the conclusions of this article will be made available by the authors, without undue reservation.

## Ethics statement

The studies involving human participants were reviewed and approved by Faculty of Medicine of University of Porto's Ethics Committee (Ref 184/2020 on May 7, 2020). The patients/participants provided their written informed consent to participate in this study.

## Author contributions

ID and CS contributed to conception and design of the study, project administration—supervision, and coordination. AT and LC organized the database and performed the statistical analysis. CS and AR wrote the first draft of the manuscript, manuscript preparation, manuscript revision, reviewing, editing, and manuscript final version approval. All authors were involved in the data collection, contributed to manuscript revision, read, and approved the submitted version.

## Funding

This work was supported by national funds through the FCT-Fundação para a Ciência e a Tecnologia, I.P., within CINTESIS, R&D Unit (reference UIDP/4255/2020) and FCT, under Grant (UIDB/05198/2020; Center for Research and Innovation in Education, inED). AR is supported by FCT through the Ph.D. grants: SFRH/BD/145253/2019.

## Conflict of interest

The authors declare that the research was conducted in the absence of any commercial or financial relationships that could be construed as a potential conflict of interest.

## Publisher's note

All claims expressed in this article are solely those of the authors and do not necessarily represent those of their affiliated organizations, or those of the publisher, the editors and the reviewers. Any product that may be evaluated in this article, or claim that may be made by its manufacturer, is not guaranteed or endorsed by the publisher.
